# Seafarer citizen scientist ocean transparency data as a resource for phytoplankton and climate research

**DOI:** 10.1371/journal.pone.0186092

**Published:** 2017-12-06

**Authors:** Secchi Disk Seafarers, Samantha Lavender, Gregory Beaugrand, Nicholas Outram, Nigel Barlow, David Crotty, Jake Evans, Richard Kirby

**Affiliations:** 1 Secchi Disk Seafarers, Citizen scientist participants in the Secchi Disk study, The oceans; 2 Pixalytics Ltd, Plymouth, United Kingdom; 3 CNRS, University of Lille, University Littoral, Wimereux, France; 4 Plymouth University, Plymouth, United Kingdom; 5 The Secchi Disk Foundation, Plymouth, United Kingdom; Technical University of Denmark, DENMARK

## Abstract

The oceans’ phytoplankton that underpin the marine food chain appear to be changing in abundance due to global climate change. Here, we compare the first four years of data from a citizen science ocean transparency study, conducted by seafarers using home-made Secchi Disks and a free Smartphone application called Secchi, with contemporaneous satellite ocean colour measurements. Our results show seafarers collect useful Secchi Disk measurements of ocean transparency that could help future assessments of climate-induced changes in the phytoplankton when used to extend historical Secchi Disk data.

## Introduction

The ocean’s phytoplankton accounts for approximately 50% of global net primary production [1,2] and underpins the marine food chain [3], it also affects the optical and thermal sea surface properties [4,5], provides the World with oxygen [6], and plays a central role in the global carbon cycle [7]. Living near the sea surface, the phytoplankton are sensitive to the varying environmental regime, which is now also altering due to climate change [8]. In a study of a 100-year trend in global upper-ocean phytoplankton chlorophyll concentrations, Boyce et al. [9] reported a 40% decline in global phytoplankton primary production since 1950; they proposed that water column mixing and the supply of growth-promoting nutrients to the sea surface had reduced due to warming global temperatures. The study [9] attracted criticism however, as the analysis combined two methods for measuring phytoplankton chlorophyll to achieve a 100-year global dataset [10,11]; Secchi Disk ocean transparency measurements were the dominant data in the first 50 years of the time series whereas colorimetric measurements were more popular in recent decades. Also, different phytoplankton abundance data indicated that phytoplankton had increased in the North Atlantic and North Pacific over the same period [12].

Recent analyses of short-term change in sea surface phytoplankton, determined from satellite ocean colour measurements over the last 30 years provide further evidence of climate effects on the phytoplankton. Rousseaux and Gregg [13] and Roxy et al. [14] have reported global declines in diatoms and declines in phytoplankton chlorophyll in the Indian Ocean, respectively. Estimating phytoplankton change from satellites has its own difficulties however, including how to both account for an uneven distribution of phytoplankton abundance throughout the water column [15] and the phytoplankton’s acclimation to different light intensities [16].

The only way to assess long-term, global, ocean phytoplankton change in the water column from the beginning of the 20^th^ Century, without combining different types of data, is to increase present-day Secchi Disk ocean transparency measurements to enable a direct future comparison with historical Secchi Disk data. While there are more professional marine biologists than ever before, few go to sea and still fewer go far offshore, and open-ocean research cruises rarely return to the same location. In contrast, many of the public go to sea regularly for recreation or work and often to a similar place, and ocean passages sailed by cruisers follow similar routes dictated by the time of year and favourable prevailing winds [17]. It is to increase the present-day measurement of ocean transparency data that the seafarer, citizen science Secchi Disk study began in 2013 (http://www.secchidisk.org); the study enables any seafarer equipped with a Secchi Disk and a free Smartphone application called Secchi to measure ocean transparency and submit it to a database.

Here, we compare the first 4 years of *in situ* ocean transparency measurements collected by seafarers to satellite estimated chlorophyll and Secchi depth values. While a comparison to contemporaneous satellite data will be imperfect because the biological and environmental vertical and horizontal heterogeneity is simplified by satellite remote sensing, which provides an integrated value [15,16], the comparison provides an opportunity to assess the seafarers’ citizen science Secchi depth measurements, and demonstrates their potential usefulness for expanding the historical Secchi Disk data to understand the effects of climate change on the oceans’ phytoplankton.

## Materials and methods

### The Secchi Disk

**Fig 1:**
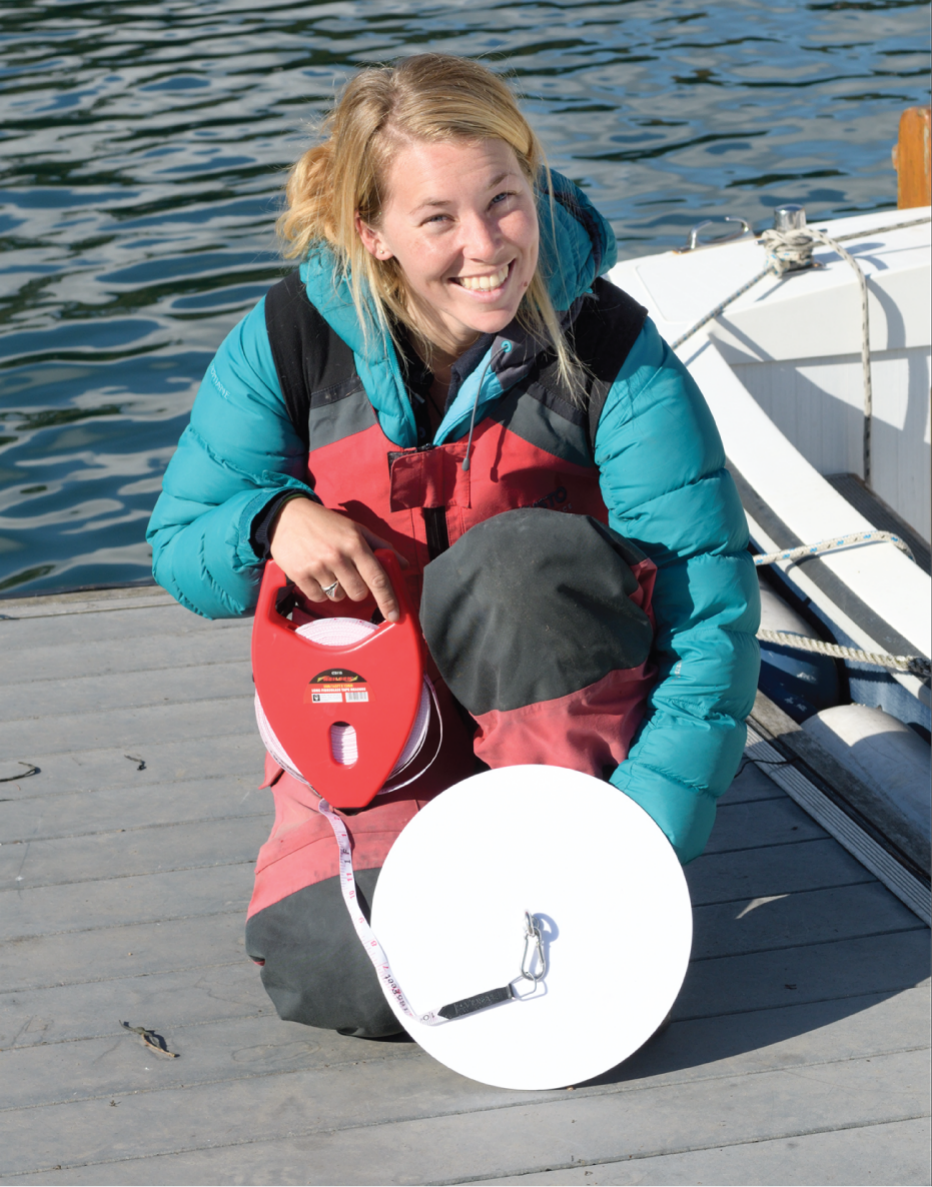
Seafarer citizen scientist and yachtswoman susie goodall with a 30 cm diameter Secchi Disk and Fibreglass tape measure.

A marine Secchi Disk is a white, 30 cm diameter disk attached to a marked line or Fibreglass tape measure and weighted from below so the disk sinks vertically into the seawater (Fig 1, the individual in this manuscript has given written informed consent (as outlined in PLOS consent form) to publish these case details.); its method of use, optical properties, limitations and variability are well understood [18,19] and are taken into account in the instructions provided to seafarers in the free Smartphone application called Secchi. When a Secchi Disk is lowered by a person into the sea from a boat the depth (m) beneath the sea surface at which the Secchi Disk just disappears from sight is called the Secchi depth, and this measures ocean transparency. The major influences upon the transparency of seawater in the open-ocean are phytoplankton pigments such as chlorophyll and their breakdown products; resuspended sediments and dissolved organic matter from rivers further reduce transparency and introduce optical errors in bays and when bathymetry < 25 m and the distance is < 1 km from shore [9]. Consequently, in the open-ocean, phytoplankton chlorophyll correlates with absorption and diffuse light attenuation and therefore the Secchi depth decreases as phytoplankton chlorophyll increases [19]. The Secchi Disk’s low cost, easy manufacture and simple use make it a suitable citizen science tool for measuring ocean transparency and so also the phytoplankton in the open ocean.

### The Secchi Disk study and the free Secchi Smartphone application

The Secchi Disk study [20] began in February 2013 when the website and the free Secchi Smartphone applications were released. How to make and use a Secchi Disk (what time of day to take a measurement, the position of the sun with respect to the observer, and amount of cloud cover) are described in the Secchi application. The Secchi application is available for iOS and Android operating systems as a native application, and as a web application running within the Smartphone’s web browser for the Windows operating system. To record a Secchi depth the seafarer first uses the Secchi application to obtain a GPS location, date and time using the Smartphone’s inbuilt GPS receiver. The seafarer then records the Secchi depth (m) using their Secchi Disk and inputs this value into the application. The Secchi application can store multiple records that the user uploads to an online database when they are next connected to a network; an interactive, mapped visualisation of the data is publicly available from the Secchi Disk study website. The Secchi Disk data used in this study are given in S1 Table. The Secchi application also allows the user to provide optionally, some additional observations, which include the sea temperature, a short text note, and a photograph if the Smartphone has a camera (the photograph option is unavailable in the Secchi Web application).

### Satellite and seafarer Secchi depth data

Global sea surface chlorophyll *a* concentrations [mg/m^3^] between February 2013 and January 2017 were obtained from the Moderate Resolution Imaging Spectroradiometer (MODIS)-Aqua global Level-3 8-day and 1-day temporal composites (4 by 4 km^2^ pixel resolution) [21], where chlorophyll *a* is calculated using an algorithm called the Ocean Color Index (OCI) [22]. 8-day temporal composites were used to maximise the number of seafarer Secchi depth data matchups, while the 1-day composites were used to determine the influence of temporal smearing.

The seafarer Secchi depth data were matched to a single pixel and extracted for pixels containing valid values; a 3 by 3 pixel kernel extraction was also investigated, but suffered from spatial smearing. The chlorophyll *a* concentrations were used to estimate Secchi depths using the Morel et al. [23] algorithm, which is designed for open-ocean waters dominated by phytoplankton. Morel et al. [23] used an iterative process to estimate *in-situ* Secchi depth from *in-situ* chlorophyll *a* concentrations, with the equation that best fitted this relationship being the algorithm. Validation using MODIS Level-3 composite data and Secchi disk measurements (available from United States National Oceanographic Data Center) resulted in adjusted coefficients as the modelled values tended to exceed the measured values [23], and it is these adjusted coefficients that have been used for this implementation.

### Bathymetry and distance from the nearest coast data

Bathymetry data were obtained from a global ocean bathymetry chart (0.1 latitude by 0.1 longitude) [24]. The distance from the nearest coast was obtained from a global data set of distances from the nearest coastline estimated at a spatial resolution of 0.01 degrees [25]. Data were interpolated using the inverse-squared distance method to give a bathymetry or distance from the nearest coast for each Secchi Disk sample. We then examined the influence of either bathymetry or distance from the nearest coast on the relationship between the Secchi depth estimated from the MODIS-Aqua Level-3 8-day composite chlorophyll *a* data and seafarer Secchi depths.

## Results

**Fig 2:**
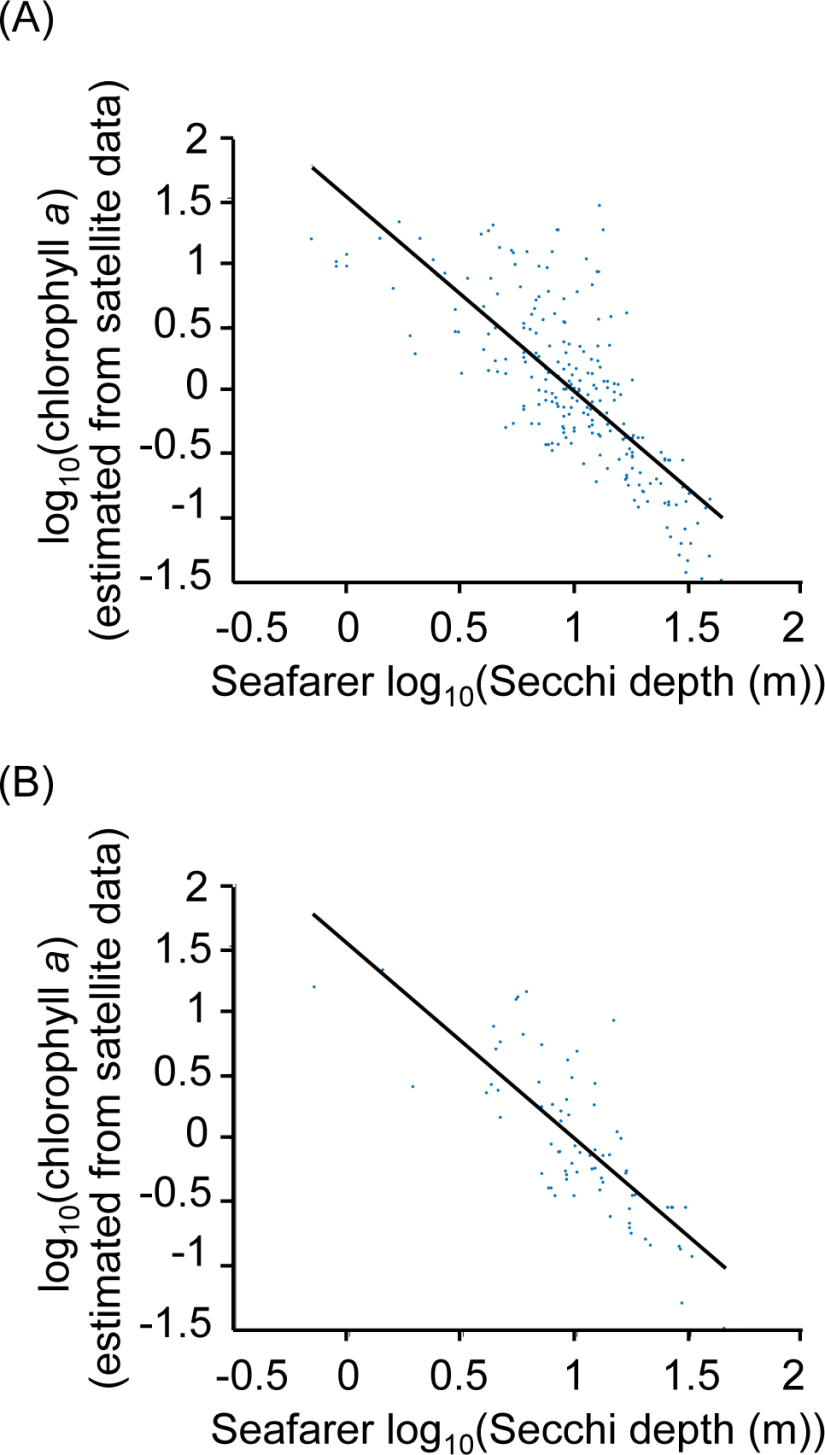
Citizen science Secchi depth versus extracted MODIS-Aqua Level-3 log_10_(chlorophyll *a*) data, available at 4 by 4 km resolution. (A) MODIS-Aqua Level-3 8-day temporal composite log_10_(chlorophyll *a*) data. (B) MODIS-Aqua Level-3 1-day temporal composite log_10_(chlorophyll *a*) data.

**Fig 3:**
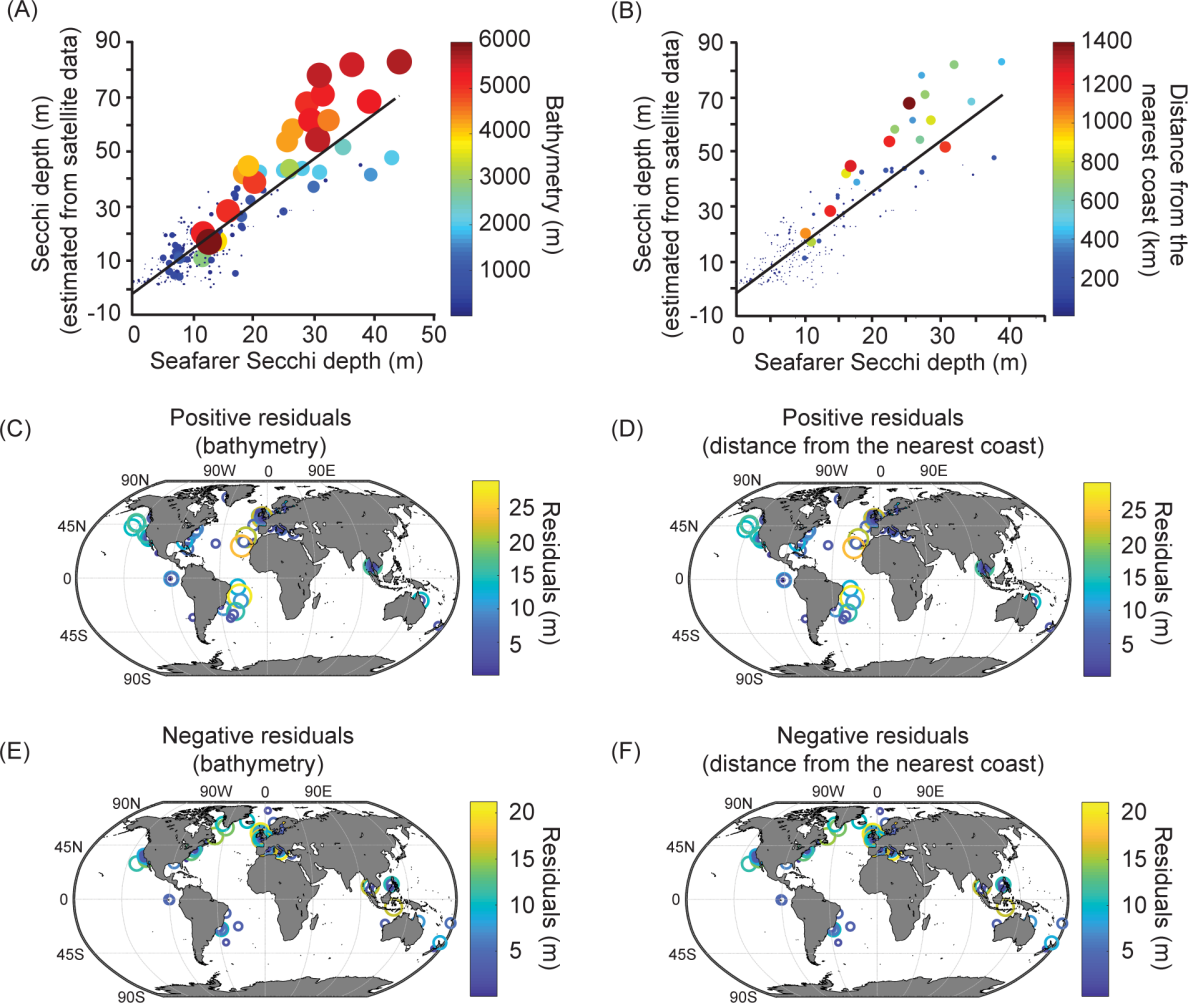
Seafarer citizen science Secchi depths, Secchi depths estimated from satellite ocean colour data, and the influence of bathymetry or distance from the nearest coast. (A) Seafarer Secchi depth data and Secchi depth estimated from the MODIS-Aqua Level-3 8-day temporal composite chlorophyll *a* data, and bathymetry and (B) with distance from the nearest coast; the size and colour of the points are proportional to the bathymetry or the distance from the coast, respectively. The black line originates from a linear regression analysis. (C) Positive and (E) negative residuals for the relationship in Fig 3A. (D) Positive and (F) negative residuals for the relationship in Fig 3B.

Fig 2A and 2B shows the similar relationship we obtained between the seafarer citizen science log_10_(Secchi depth) data and the extracted MODIS-Aqua 8-day and 1-day temporal composite chlorophyll *a* data, respectively (*r* = -0.76, p<0.01 and *r* = -0.79, p<0.01 for the 8-day and 1-day data, respectively). In both cases, when sea surface chlorophyll *a* is higher, the Secchi depth is more variable and conversely when sea surface chlorophyll *a* is low. As the 8-day temporal composite and 1-day temporal composite data gave similar results we used the former for subsequent analyses in order to provide the largest data set. Fig 3A and 3B shows the strong positive correlation (*r* = 0.86, p<0.01) between the seafarer citizen science Secchi depth data and the Secchi depth estimated from the MODIS-Aqua chlorophyll *a* 8-day temporal composite data, and the similar influences of either bathymetry or the distance from the nearest coast on the relationships, respectively. Linear regression analysis after removing negative values (*b* = 1.64, 95% CI 1.51–1.76, p<0.01) indicates satellite ocean colour measurements are generally 1.6 times greater than the seafarer Secchi depth measurements; for example when the Secchi Disk measures 10 m, the satellite-derived Secchi depth estimation is 14.52 m (13.28–15.77 m, 95% confidence interval) and over the open-ocean, especially, ~30 m in some places in the Atlantic and Pacific (Fig 3C and 3D). Over coastal regions, both positive and negative residuals occur (Fig 3C–3F).

## Discussion

Our study shows that ocean transparency data collected by seafarer citizen scientists agrees well with satellite chlorophyll *a* and satellite Secchi estimates. As expected however, the seafarer Secchi depth data shows scatter due to both biological variability and non-biological factors affecting water column transparency. Fig 2A and 2B show the relationship between satellite data and seafarer Secchi depths was unaffected by the temporal resolution. Previous research indicates that the satellite Secchi depth estimate over the open-ocean overestimates the measured Secchi depth because it assumes the layer extending down to the Secchi depth is homogenous, whereas chlorophyll *a* always varies with depth [23]; chlorophyll levels are typically lower at the surface than at depth under stratified open-ocean conditions and so satellite chlorophyll underestimates the depth-averaged value. In addition, globally applicable satellite algorithms are primarily designed for open-ocean waters, which may also help to explain scatter at small seafarer Secchi depths in Fig 2 (where there are likely to be abiological factors) and the distribution of residuals in Fig 3C–3F (primarily positive residuals in the open-ocean and negative residuals in coastal waters). There has also been an ageing of the MODIS-Aqua sensor, which has affected the derived chlorophyll *a* from 2013 onwards, but using the latest reprocessing dataset minimises this degradation in accuracy. In the longer term the aim is to use merged satellite ocean-colour datasets to minimise the dependency on specific satellite sensors. For the above reasons our results are within the level of expected agreement between Secchi Disk readings and satellite estimates of chlorophyll *a* and Secchi depth; even for chlorophyll *a*, satellite estimates target an accuracy of 30% [26] and are known to struggle to reach this in all but open-ocean waters. Future research will test a broader range of satellite algorithms, so that the satellite estimate of the Secchi depth is more accurate.

Secchi Disk ocean transparency data can therefore help us understand *in situ* versus satellite observations of phytoplankton (Figs 2 and 3A). The alternative to citizen science data collected from the marine environment is data collected by scientists, but their use of Secchi Disks has declined in recent years [27]. As participation by seafarer citizen scientists using Secchi Disks increases their data can contribute towards understanding how global measures of ocean transparency and phytoplankton are changing over long-term timescales from the beginning of the 20^th^ century, and so hopefully, address the study of Boyce et al. [9].

There are over 7 billion people on Earth whose activities are causing environmental change and putting pressure on finite ecosystems [28]. About 70% of the world population lives within 60 km of the shoreline, and we catch around 80 million tonnes of fish every year. Between 24.2–35.3% of the primary production of tropical and non-tropical continental shelf ecosystems supports their industrialised, shelf fisheries, respectively [29]. Citizen science, especially of the plankton, therefore has another benefit to data collection that is to raise the general public’s understanding of the natural world through their active engagement in its study, which can only help towards environmental awareness and a sustainable future.

## Supporting information

S1 Table Seafarer Secchi depth data (m), location (latitude and longitude), date collected (day of year and year) and data ID. (CSV)
